# Zinc gluconate ameliorates antibiotics/PGN-induced intestinal injury via modulating the gut barrier, macrophages immunity and gut microbiota

**DOI:** 10.3389/fmicb.2026.1791355

**Published:** 2026-08-20

**Authors:** Xia Yang, Sumei Wei, Shiqi Su, Ying Su, Liancheng Lan, Xiuqi Chen, Ting Huang, Qingwen Shan

**Affiliations:** 1 Department of Pediatrics, The First Affiliated Hospital of Guangxi Medical University, Nanning, China; 2 Difficult and Critical Illness Center, Pediatric Clinical Medical Research Center of Guangxi, The First Afflicted Hospital of Guangxi Medical University, Nanning, China; 3 Guangxi Key Laboratory of Aquatic Genetic Breeding and Healthy Aquaculture, Guangxi Academy of Fishery Sciences, Nanning, China

**Keywords:** antibiotic-associated colitis, gut microbiota, macrophage, peptidoglycan, zinc gluconate

## Abstract

**Objective:**

This study aimed to investigate the protective effects of zinc gluconate (ZnG) against antibiotics and peptidoglycan (PGN) induced intestinal injury by targeting gut barrier integrity, macrophage, and gut microbiota homeostasis.

**Methods:**

Twenty four C57BL/J mice were randomly divided into four groups: normal group (NC), antibiotic cocktail group (ABX), model group (ABX+PGN), and ZnG treatment group (ZnG+ABX/PGN). An antibiotic associated dysbiosis model was established via oral gavage of an antibiotic cocktail, followed by intraperitoneal injection of PGN (10 mg/kg) to simulate Gram positive (G^+^) bacteria associated challenge in antibiotic associated colitis (AAC) after microbiota disruption. Subsequently, ZnG was administered for therapeutic intervention. Parameters assessed included general condition of mice, intestinal permeability, barrier integrity, macrophage polarization, and gut microbiota composition.

**Results:**

The results indicated that ZnG significantly increased the body weight of the mice treated with ABX and PGN. It also markedly alleviated pathological and ultrastructural damage in the colon. ZnG improved intestinal permeability by reducing serum diamine oxidase (DAO) levels and restored intestinal integrity by upregulating tight junction proteins (TJs), including zonula occludens-1 (ZO-1), Occludin, and mucin 2 (MUC2). In addition, ZnG modulated the macrophage immunity by regulating the expression of polarization associated proteins (iNOS/ARG-1) and inhibited activation of the TLR2-NF-κB signaling pathway. Microbiota analysis revealed that ZnG increased gut microbial diversity while modulating the microbial community structure and its predicted functional potential in mice.

**Conclusions:**

These findings demonstrate that ZnG exhibits therapeutic potential for AAC through modulation of gut microbiota, enhancement of gut barrier, and regulation of immune responses.

## 1 Introduction

The widespread use of antibiotics frequently disrupts intestinal homeostasis, which is maintained by coordinated mechanical, immune, and microbial components (Massier et al., 2021; Patangia et al., 2022). This disruption creates ecological niches for opportunistic pathogens and can trigger antibiotic associated colitis (AAC) (Wei et al., 2026), which has emerged as a major challenge in clinical practice (Wang et al., 2020a; Kwon et al., 2022). *Staphylococcus aureus* (*S. aureus*) was among the earliest opportunistic pathogens linked to AAC (Niamba et al., 2025). Although *Clostridioides difficile* infection (CDI) has long been regarded as the principal cause of antibiotic associated diarrhea (AAD) (Shirley et al., 2023), it accounts for only approximately 25% of cases (Barbut and Meynard, 2002), suggesting that Gram positive (G^+^) bacteria, including *S. aureus*, may be underrecognized contributors. Peptidoglycan (PGN), a conserved G^+^ cell wall component, activates the TLR2/NF-κB signaling pathway, thereby promoting inflammation and compromising barrier integrity (Bersch et al., 2021; Sun et al., 2022a). Simultaneously, lamina propria macrophages are primary regulators of intestinal inflammation, and their M1/M2 polarization shapes disease progression and host outcomes (Locati et al., 2020; Wang et al., 2020b). Collectively, dysbiosis, barrier impairment, and aberrant macrophage polarization may synergistically drive AAC pathology.

Given the multifactorial nature of AAC pathogenesis, nutritional and trace element based interventions have attracted increasing attention as potential modulators of intestinal homeostasis. Among them, zinc is an essential trace element with fundamental roles in growth, enzymatic activity, and immune regulation (Stiles et al., 2024). Supported by strong clinical evidence, zinc supplementation has been incorporated into multiple international guidelines as a key preventive and therapeutic strategy for pediatric diarrheal diseases (Ali et al., 2024; Kundu et al., 2025). However, its biological effects are closely associated with its chemical form and bioavailability. Previous studies have demonstrated that organic formulations such as zinc glycinate and zinc gluconate (ZnG) and chelated zinc formulations, may exhibit higher bioavailability compared with inorganic zinc salts, including zinc oxide (ZnO) and zinc sulfate (Wegmüller et al., 2014; Joshua Ashaolu et al., 2023; Piacenza et al., 2023). These advantages may be attributed to enhanced solubility to maintain zinc in a more absorbable form during gastrointestinal transit, thereby facilitating intestinal uptake (Wegmüller et al., 2014).

Previous studies have shown that alterations in zinc homeostasis can significantly affect both the composition and function of the intestinal microbiota. Under zinc deficient conditions, microbial α and β diversity are reduced (Gaulke et al., 2018; Chen et al., 2021), accompanied by decreased production of short chain fatty acids (SCFAs) and alterations in microbial metabolic functions (Reed et al., 2015), thereby increasing susceptibility to intestinal injury. Conversely, zinc supplementation can reshape the gut microbial ecosystem under various physiological and pathological conditions. For instance, ZnO has been reported to suppress enteric pathogens such as *Clostridium* and *Cronobacter* (Sun et al., 2022b), while appropriate zinc supplementation can remodel gut microbial communities and enrich commensal taxa under specific physiological or pathological conditions (Wong et al., 2025). Overall, zinc homeostasis is closely linked to gut microbial balance.

Despite this potential, effective clinical strategies that directly promote intestinal barrier repair in gut injury diseases remain limited (Fortea et al., 2021; Kong et al., 2024). Importantly, accumulating evidence from animal models indicates that zinc homeostasis plays a critical role in maintaining intestinal barrier integrity. For example, marginal or dietary zinc deficiency has been shown to aggravate intestinal barrier dysfunction, increase epithelial permeability, and exacerbate inflammatory responses in *Enterotoxigenic Escherichia coli* induced injury mouse models (Wang et al., 2022). Similarly, mice fed zinc depleted diet exhibit disrupted junction stability and intestinal barrier function (Higashimura et al., 2020). In contrast, zinc glycinate and zinc carnosine improve intestinal barrier integrity in models of inflammatory bowel disease (IBD) and heat stress (Odashima et al., 2006; Zulfiqar et al., 2025). Consistently, our previous work demonstrated that ZnG markedly attenuates LPS induced intestinal injury under dysbiosis conditions by restoring tight junction (TJ) protein expression (Wang et al., 2024). These findings provide a support for considering zinc as a candidate intervention for barrier repair in intestinal injury settings. However, the therapeutic potential of ZnG against other pathogenic components such as PGN remains unknown. Therefore, evaluating whether ZnG can mitigate ABX and PGN induced mucosal injury is crucial to demonstrating its protective spectrum against diverse microbial challenges.

Beyond its effects on the microbiota and epithelial barrier, zinc is also an essential regulator of innate and adaptive immune homeostasis. Previous studies have demonstrated that zinc deficiency impairs neutrophil chemotaxis, disrupts T cell maturation, and causes dysregulated cytokine production, thereby exacerbating infection and inflammatory responses (Wessels et al., 2021). Within the innate immune system, zinc regulates multiple aspects of macrophage function, enhancing phagocytic activity and bacterial clearance in sepsis models (Nowak et al., 2012) and suppressing macrophage pyroptosis while promoting anti-inflammatory phenotypes via regulation of intracellular zinc pools (Hu et al., 2025). Zinc has also been implicated in dampening inflammatory signaling through TLR/NF-κB pathways (Chang et al., 2024). Collectively, these findings suggest that regulation of macrophage function may represent an important mechanism underlying the intestinal protective effects of zinc.

In this study, we employed a murine model of AAC induced by PGN following antibiotic mediated dysbiosis to simulate G^+^ bacterial bacteria associated challenge. We aimed to investigate the protective potential and underlying mechanisms of ZnG in this model, with a specific focus on its roles in restoring intestinal barrier integrity, modulating macrophage mediated immune responses, and partial modulating the gut microbiota. Our findings provide novel insights into the therapeutic application of ZnG as a targeted strategy for repairing intestinal injury in AAC.

## 2 Materials and methods

### 2.1 Laboratory animals

Twenty four male C57BL/6J mice (5–6 weeks old; 20 ± 2 g) were purchased from Guangdong Vital River Laboratory Animal Technology Co., Ltd (Guangzhou, China). All mice were kept under standardized housing conditions throughout the study with specific pathogen free systems, ambient temperature at 22 °C−25 °C, relative humidity of 45%−55%, and a 12 h light/dark cycle. Throughout the study, mice were housed in clean cages with *ad libitum* access to food and water. All animal procedures were approved by the Ethics Committee of Guangxi Medical University (approval number: 202210015) and performed in accordance with institutional guidelines.

### 2.2 Animal experimental protocol

**Figure 1:**
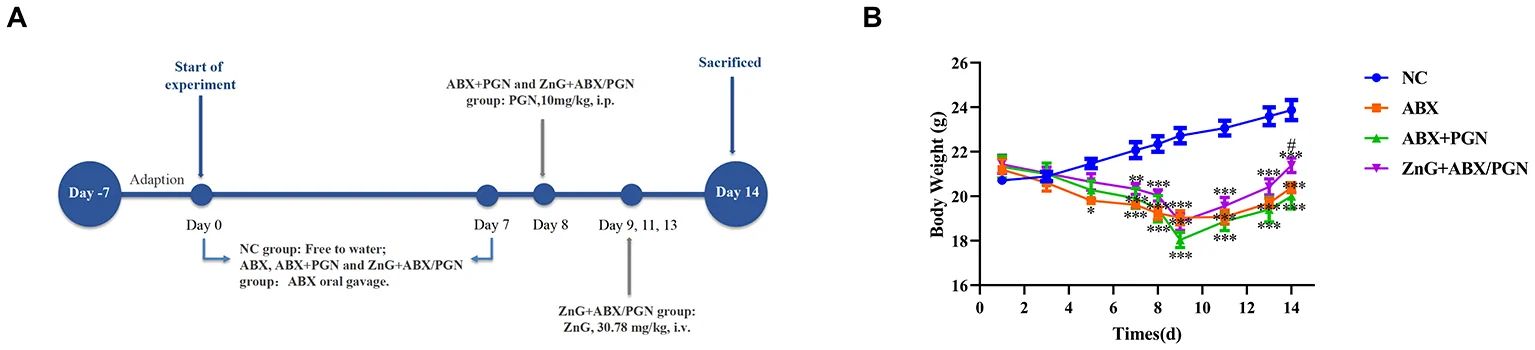
Experimental design and effects of ZnG on body weight changes in ABX and PGN induced mice. **(A)** Schematic timeline of the experiment. **(B)** Body weight changes over time in each group. All data are presented as mean ± SEM (*n* = 5-6 mice per group). Compared with group NC: *p < 0.05, **p < 0.01, ***p < 0.001; Compared with group ABX+PGN:#*p*<0.05.

Mice were acclimated for 1 week and then randomly assigned to four groups (*n* = 6): normal control group (NC), antibiotic cocktail group (ABX), model group (ABX+PGN), and ZnG treatment group (ZnG+ABX/PGN). The NC group received a standard diet and water, whereas all other groups were administered an antibiotic cocktail (ABX) by oral gavage once daily for seven consecutive days. The ABX formulation was prepared as previously described in our earlier studies (Wang et al., 2024) and consisted of ampicillin (1 mg/mL, A9518-25G-9), metronidazole (1 mg/mL, M1547-25G), gentamicin (1 mg/mL, E003632-1 g), neomycin sulfate (1 mg/mL, N6386-5G), and vancomycin hydrochloride (0.5 mg/mL, 1404-93-9). On day 8, mice in the ABX+PGN and ZnG+ABX/PGN groups received an intraperitoneal injection of PGN (10 mg/kg, 77140, Sigma) to induce AAC. On days 9, 11, and 13, mice in the ZnG+ABX/PGN group were treated with ZnG (30.78 mg/kg, analytical grade, Meklin) via intravenous injection through the tail vein. Dosing regimens for the antibiotic cocktail and ZnG were based on our previous studies (Wang et al., 2024). Throughout the experiment, body weight, general condition, and fur appearance were regularly monitored. On day 14, mice were anesthetized, and blood, fecal samples and colon tissues were collected (Figure 1A). Blood samples were used for subsequent ELISA analysis, colon tissues were preserved for histological and molecular analyses, and fecal samples were used for 16S rRNA analysis of gut microbiota.

### 2.3 Hematoxylin and eosin (H&E) staining

Colon tissues were fixed in 4% paraformaldehyde (Servicebio, Wuhan, China) for 24 h at room temperature, dehydrated through a graded ethanol series using a rotary microtome (Leica RM2016, Wetzlar, Germany), and embedded in paraftin. Paraffin embedded tissues were sectioned at a thickness of 4 um. Tissue sections were mounted on glass slides and dried at 60 °C for 2 h. For HE staining, sections were deparaffinized in xylene and rehydrated through a descending ethanol gradient to distilled water. Sections were stained with hematoxylin for 3–5 min, differentiated in acid alcohol, rinsed in running tap water, and counterstained with eosin for 1–2 min. After dehydration and clearing, slides were sealed with neutral resin. Then colonic histological architecture was examined under a microscope (Nikon, Tokyo, Japan), and representative images were captured.

### 2.4 Transmission electron microscopy (TME) assays

Colon tissues were cut into approximately 1 mm^3^ fragments and immediately fixed in 2.5% glutaraldehyde for 2 h. Samples were rinsed 3 times with phosphate buffered saline and post fixed in 1% osmium tetroxide for 2 h at room temperature in the dark. Tissues were then dehydrated through a graded ethanol series, embedded in epoxy resin, and polymerized to form resin blocks. Ultrathin sections (~60 nm) were prepared using an ultramicrotome and sequentially stained with 2% uranyl acetate and 2.6% lead citrate. After washing and air drying, sections were examined under a transmission electron microscope (Hitachi H-7650, Tokyo, Japan). Image acquisition was performed by an investigator blinded to the experimental group.

### 2.5 Immunohistochemical (IHC) assays

Paraffin embedded tissue sections were deparaffinized and treated with 3% hydrogen peroxide to quench endogenous peroxidase activity, followed by antigen retrieval in 0.01 mol/L citrate buffer (pH 6.0). Sections were then blocked with goat serum at room temperature for 30 min and incubated overnight at 4 °C with primary antibodies against MUC2 (1:400, 27675-1-AP, Proteintech), inducible nitric oxide synthase (iNOS; 1:400, 18985-1-AP, Proteintech), and arginase-1 (ARG-1; 1:400, 16001-1-AP, Proteintech). After incubation with primary antibodies, sections were incubated with secondary antibody (1:10000, 80008-1-RR, Proteintech). Immunoreactivity was visualized using 3,3′-diaminobenzidine (DAB) substrate kit (Solarbio, Beijing, China), followed by hematoxylin counterstaining. Sections were then dehydrated through a graded ethanol series and mounted with neutral resin. Positive staining was identified by the presence of light brown granules in the cytoplasm. Three randomly selected microscopic fields per section were used for quantitative analysis, and the average value for each mouse was used for statistical analysis. Images were captured under an optical microscope, and ImageJ software (v1.8.0, NIH, USA) was used for semi-quantitative analysis of MUC2, iNOS, and ARG-1 expression. A fixed threshold was applied based on negative controls. The average optical density (AOD) was calculated as integrated optical density (IntDen) divided by Area.

### 2.6 Analysis of intestinal permeability

Mouse blood samples were centrifuged at 1,000 × g for 15 min to obtain serum. Diamine oxidase (DAO) levels were measured using a commercial biochemical assay kit (FineTest, Wuhan, China) according to the manufacturer's instructions. Briefly, serum samples from individual mice and standards (100 μL/well) were added to 96-well plates precoated with anti-DAO antibody and incubated at 37 °C for 90 min. Then added 100 μL biotin-labeled antibody working solution into each well and incubated at 37 °C for 60 min. After washing the plates 3 times with wash buffer, 100 μL of HRP-conjugated detection antibody was added to each well and incubated at 37 °C for 30 min, and wash 5 times again. Subsequently, 90 μL of TMB substrate solution was added and incubated in the dark at 37 °C for 15 min, followed by the addition of 50 μL stop solution. The optical density (OD) values were detected at 450 nm using a Multiskan Go microplate reader (Thermo Fisher Scientific, USA). A standard curve was generated with CurveExpert 1.4 software to calculate DAO concentrations.

### 2.7 Western blot analysis

Colon tissues were lysed in RIPA buffer supplemented with protease and phosphatase inhibitors and homogenized. Lysates were centrifuged, and the supernatants were collected for total protein extraction. Protein concentration was measured using a bicinchoninic acid assay. Protein samples were denatured by boiling, separated by sulfate polyacrylamide gel electrophoresis, and transferred onto polyvinylidene fluoride membranes. Membranes were blocked with 5% bovine serum albumin and incubated overnight at 4 °C with primary antibodies against ZO-1 (1:5000, 21773-1-AP, Proteintech), Occludin (1:10000, 27260-1-AP, Proteintech), p65 (1:3000, 10745-1-AP, Proteintech), and TLR2 (1:800, WL01747, Wanleibio). After washing with Tris-buffered saline containing Tween 20, membranes were incubated with HRP-linked secondary antibody (1:10000, R511203, Zenbio) for 2 h at room temperature. Protein bands were visualized using an enhanced chemiluminescence detection kit (BL520A, Biosharp) and quantified with ImageJ software. Images were captured using the Touch Imager chemiluminescence imaging system (e-BLOT Life Science, Shanghai, China). β-actin (1:3000, R23613, Zenbio) was used as the internal control.

### 2.8 16S rRNA analysis of gut microbiota

Total fecal DNA was extracted from individual mouse samples using the cetyltrimethylammonium bromide method (Yu et al., 2023), and the concentration and purity of the DNA were subsequently assessed using a NanoDrop 2000 UV–Vis spectrophotometer (Thermo Fisher Scientific, Wilmington, MA, USA) in combination with 1% agarose gel electrophoresis. The distinct V3–V4 region of the bacterial 16S rRNA gene was amplified using universal the primers 338F (5′-ACTCCTACGGGAGGCAGCAG-3′) and 806R (5′-GGACTACNNGGGTATCTAAT-3′). Amplified products were verified by 2% agarose gel electrophoresis, excised, and purified using an AxyPrep DNA Gel Extraction Kit (Axygen Biosciences, Union City, CA, USA). High throughput sequencing was conducted by Wekemo Tech Group Co., Ltd. (Shenzhen, China) using the Illumina NovaSeq 6000 platform.

### 2.9 Statistical analysis

The experimental data were analyzed using GraphPad Prism 9.0 software and presented as the arithmetic mean ± standard error (mean ± SEM) for each group. Normality was first assessed using the Shapiro Wilk normality test, and homogeneity of variance was analyzed using the Brown Forsythe test. If the data followed a normal distribution and had equal variance, statistical comparisons were performed using one way analysis of variance (One way ANOVA). A *P* value of less than 0.05 was considered statistically significant.

## 3 Results

### 3.1 ZnG relieved symptoms in ABX and PGN induced mice

To assess the effects of ZnG on general clinical manifestations in mice subjected to ABX and PGN induced colitis, hair, activity, fecal traits, and body weight were monitored throughout the experiment, and body weight curves were plotted (Figure 1B). Mice in the NC group showed steady weight gain and remained clinically stable. In contrast, mice in the ABX group developed diarrhea of varying severity, accompanied by a mild loss of hair sheen, and showed a decrease in body weight (*p* = 0.0103). Compared with the ABX group, mice in the ABX+PGN group exhibited more pronounced weight loss (*p* < 0.001) and markedly aggravated diarrhea, together with disheveled hair and reduced spontaneous activity. ZnG administration partially attenuated these changes. Mice in the ZnG+ABX/PGN group showed a gradual recovery of body weight (*p* < 0.001) and a late phase improvement in clinical signs, including reduced diarrhea severity and increased activity. Notably, on day 14, body weight was significantly higher in the ZnG+ABX/PGN group than in the ABX+PGN group (*p* = 0.0476). This indicated that ZnG had a remission effect on the general condition in ABX and PGN induced mice.

### 3.2 ZnG protected the integrity of the colon in ABX and PGN induced mice

**Figure 2:**
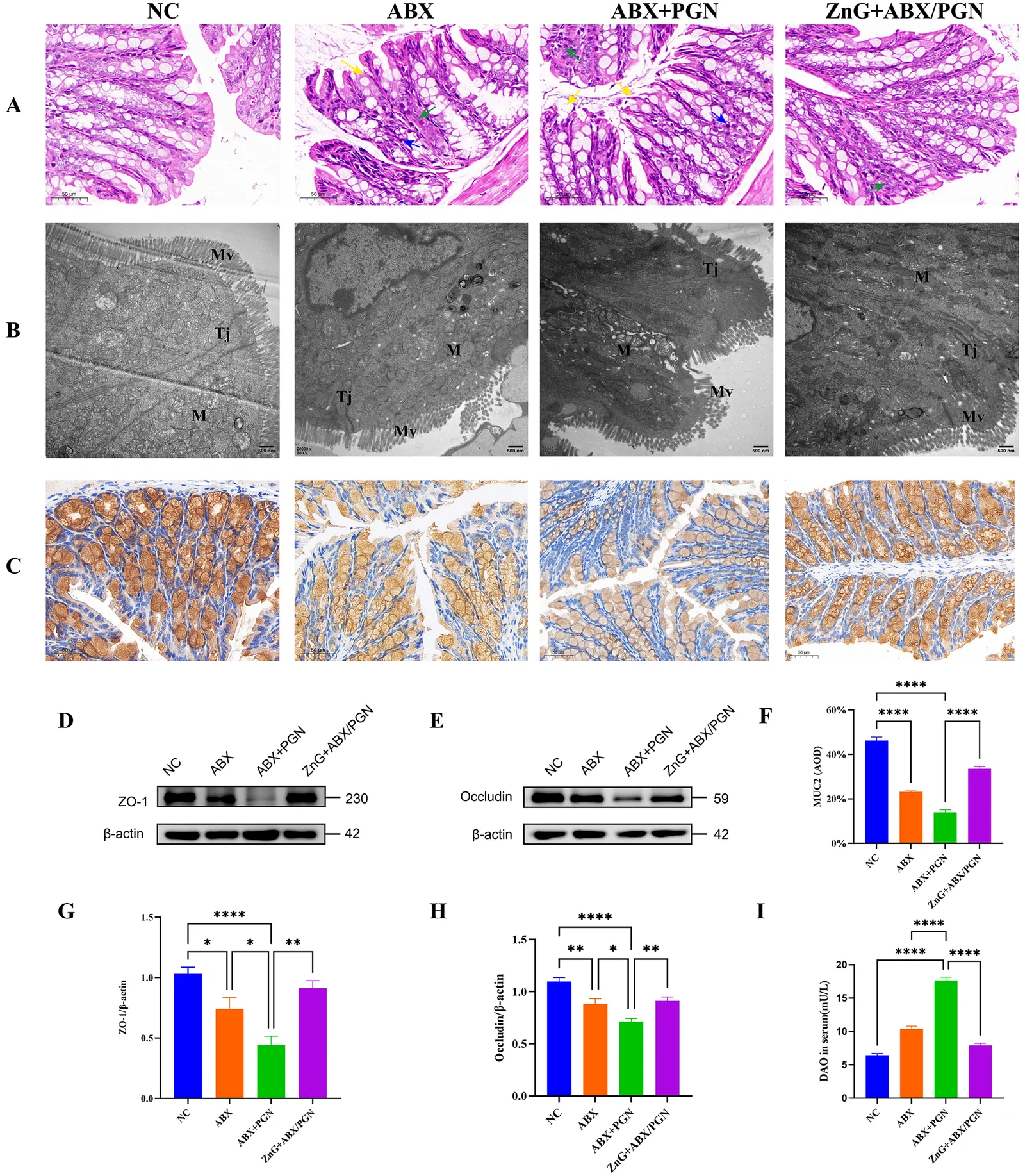
ZnG treatment ameliorates intestinal barrier damage in mice with ABX and PGN induced colitis. **(A)** H&E staining of colonic tissue (scale bar = 50 μm); **(B)** Ultrastructural changes in colonic tissue among different groups (scale bar = 500 nm), Mv, Microvilli; TJ, Tight junction; M, Mitochondria; **(C, F)** Immunohistochemistry and analysis of colonic MUC2 (); **(D, G)** Western blot and densitometric analysis of colonic ZO-1 expression; **(E, H)** Western blot and densitometric analysis of colonic Occludin expression; **(I)** Serum DAO level in each group. In (A), yellow, green, and blue arrows denote epithelial detachment, goblet cell depletion, and mucosal inflammatory infiltrates, respectively. All data are presented as mean±SEM (*n* = 5–6 mice per group) and were analyzed by One way ANOVA followed by Tukey's *post hoc* test. *p < 0.05, **p < 0.01, and ****p < 0.0001.

Histological examination revealed that the ABX and PGN challenge induced colonic tissue injury, characterized by prominent epithelial detachment, goblet cell depletion, and mucosal inflammatory infiltrates, whereas ZnG treatment mitigated these pathological changes (Figure 2A). Consistently, TEM showed that ZnG partially restored epithelial ultrastructure by preserving well organized microvilli, restoring TJ structure, and attenuating mitochondrial swelling, thereby partially mitigating colon injury (Figure 2B). IHC analysis further demonstrated that preserved MUC2 expression levels in the colon (*p* < 0.001, Figures 2C, F). Western blot results corroborated the promotion of ZO-1 (*p* = 0.0010, Figures 2D, G) and Occludin (*p* = 0.0098, Figures 2E, H) expression by ZnG. Additionally, ZnG treatment also downregulated DAO expression, with ABX and PGN exhibiting opposing effects on DAO levels (*p* < 0.001, Figure 2I). The aforementioned results indicate that ZnG effectively alleviated the damage of the intestinal barrier function in the ABX and PGN induced AAC model mice.

### 3.3 ZnG modulates macrophage balance and inhibits the TLR2/NF-κB pathway

**Figure 3:**
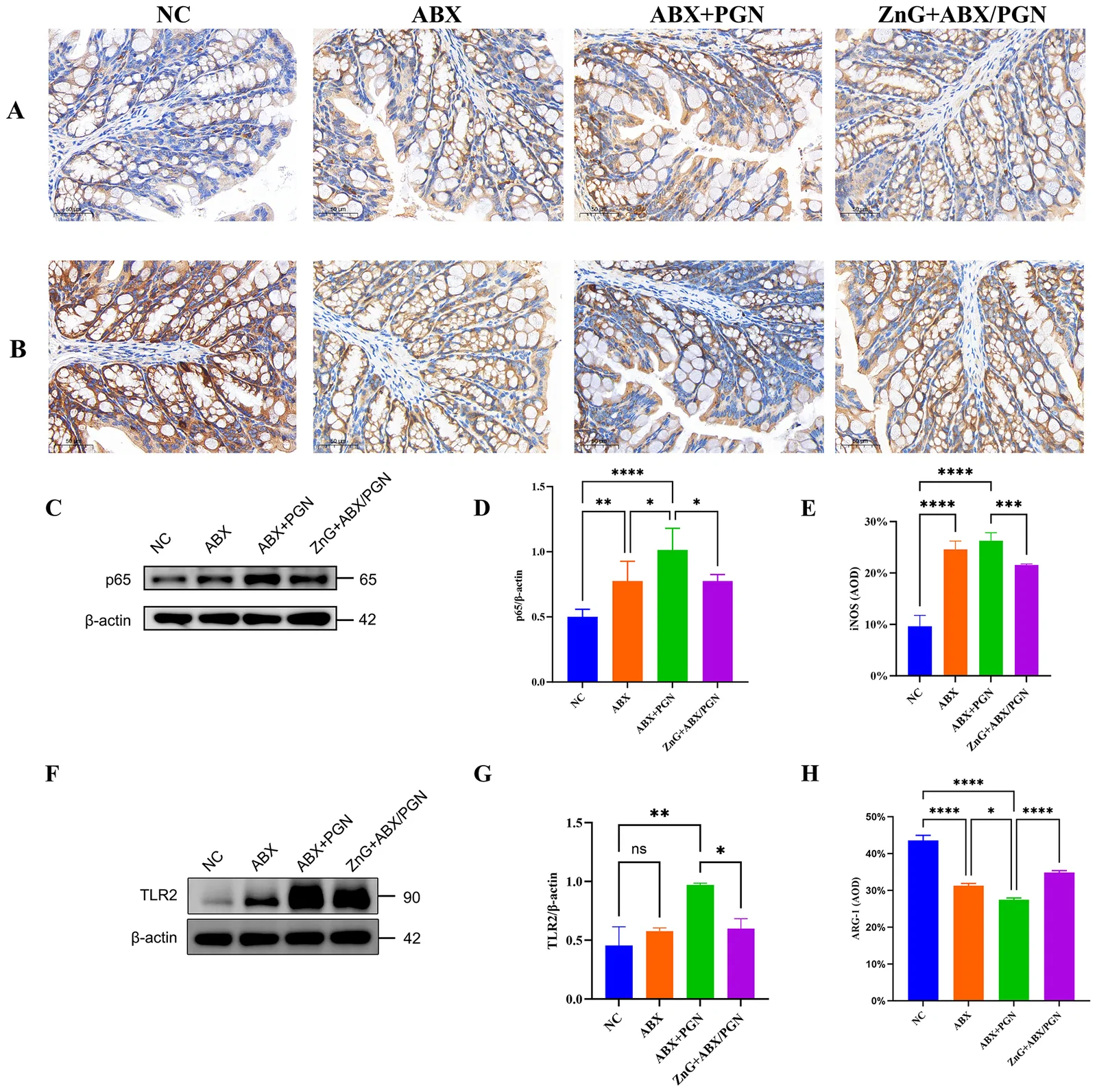
Effects of ZnG on colonic macrophages and TLR2-NF-κB signaling. **(A, B)** IHC of iNOS and ARG-1 (scale bar = 50 μm); **(C, D)** Western blot and densitometric analysis of colonic p65 expression; **(F, G)** Western blot and densitometric analysis of colonic TLR2 expression; **(E, H)** IHC analysis of iNOS and ARG-1. All data are presented as mean ± SEM (*n* = 5–6 mice per group) and were analyzed by One way ANOVA followed by Tukey's *post hoc* test. *p < 0.05, **p < 0.01, ***p < 0.001, and ****p < 0.0001.

The expression levels of the mouse colon macrophage M1 polarization marker iNOS and M2 polarization marker ARG-1 were assessed by immunohistochemistry. The positive area of iNOS in the ABX+PGN group was significantly increased compared with the NC group, but was marked attenuated after ZnG treatment (*p* < 0.001, Figures 3A, E). Conversely, the level of ARG-1 in the ZnG+ABX/PGN group was significantly higher than that in the ABX+PGN group (*p* < 0.001, Figures 3B, H). These results suggest that ZnG is associated with reduced expression of the M1 associated marker iNOS and enhanced expression of the M2 associated marker ARG-1, indicating potential alterations in macrophage phenotype. Western blot analysis was performed to determine the protein levels of TLR2 and p65 in mouse colon tissue. The results showed that the expression levels of TLR2 (*p* = 0.0433) and p65 (*p* = 0.0124) were significantly upregulated in the ABX+PGN group compared to the NC group, but were markedly decreased in the ZnG+ABX/PGN group (Figures 3C, D, F, G). These findings suggest that ZnG may alleviate colonic injury by inhibiting the TLR2/NF-κB signaling pathway.

### 3.4 ZnG altered the composition of the gut microbiota in ABX and PGN induced mice

**Figure 4:**
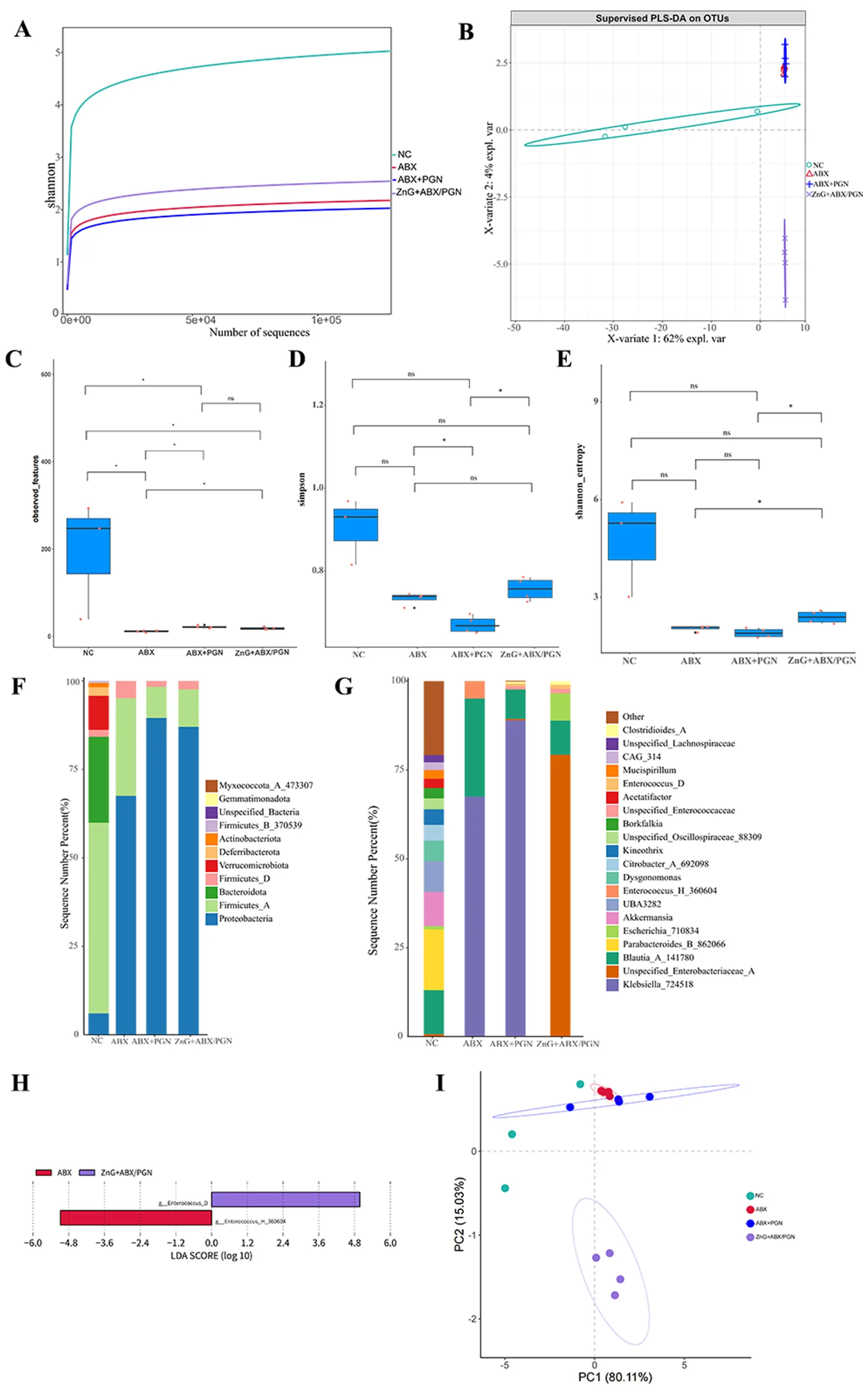
ZnG promotes gut microbiota modulation in mice with ABX and PGN induced colitis. **(A)** Rarefaction curve; **(B)** Beta diversity analysis is characterized by Partial Least Squares Discriminant Analysis (PLS-DA); Box plot of **(C)** Observed outs index, **(D)** Simpson diversity index and **(E)** Shannon index comparing gut microbial alpha diversity across experimental groups; Relative abundance of **(F)** phyla and **(G)** genera; **(H)** LEfSe analysis at genus level; **(I)** PCA plot of pathways based on the KEGG database. All data are presented as mean ± SEM (*n* = 3–4 mice per group). ns: no significance, *p< 0.05.

To elucidate whether ZnG regulates gut microbial composition, we performed 16S rRNA sequencing to analyze the gut microbiota in each group. All reads underwent quality control and length filtering. The Shannon index rarefaction curves for all groups reached a plateau as the sequencing depth increased, indicating that the sequencing depth per sample was sufficient (Figure 4A). Alpha diversity analysis revealed that, compared with the NC group, the ABX+PGN group exhibited a marked reduction in observed features (*p* = 0.05, Figure 4B). In addition, higher Shannon and Simpson indices in the ZnG+ABX/PGN group was observed (*p* = 0.029, Figures 4C, D), suggesting that ZnG treatment partially mitigated ABX and PGN induced loss of microbial diversity and richness. Consistent with these findings, partial least squares discriminant analysis (PLS-DA) revealed non-overlapping clusters for each group, indicating that ZnG intervention significantly shifted the microbial community structure (Figure 4E). Taxonomic analysis at the phylum level revealed that, compared with the NC group, the relative abundance of Proteobacteria was significantly increased in the ZnG+ABX/PGN group, whereas those of Firmicutes and Bacteroidetes were decreased (Figure 4F). Compositional analysis revealed that the abundances of *Klebsiella_724518* were significantly reduced in the ZnG+ABX/PGN group (Figure 4G). LEfSe analysis identified the *Enterococcus_D* as the biomarker for the ZnG+ABX/PGN group (Figure 4H). Principal coordinate analysis (PCoA) based on the predicted microbial functional pathway profiles revealed a distinct separation among the groups, suggesting that ZnG intervention may have modulated the functional potential of the gut microbiota (Figure 4I). Collectively, these results illustrate that ZnG can partially alleviate ABX and PGN induced gut microbiota dysbiosis, potentially contributing to protection against AAC.

## 4 Discussion

Although antibiotics are indispensable for infection control in clinical practice, prolonged or inappropriate use can markedly disturb gut microbial homeostasis and has been associated with a rising burden of AAC (Fishbein et al., 2023; Kaya et al., 2023). Evidence indicates that antibiotic exposure can disrupt the intestinal microbiota (Fishbein et al., 2023) and compromise barrier integrity, by promoting epithelial injury and perturbing junctional architecture (Sawaed et al., 2024), thereby increasing intestinal permeability and facilitating translocation of luminal bacteria, antigens, and inflammatory mediators into the lamina propria and even the systemic circulation, which in turn initiates and amplifies mucosal inflammatory and immune cascades (Wheeler et al., 2023). Zinc, an essential trace element in humans, plays a critical role in maintaining intestinal barrier integrity and modulating anti-inflammatory immune homeostasis (Hu et al., 2023; Jin et al., 2024). Notably, systemic or localized zinc deficiency is a frequent hallmark of intestinal disorders, including inflammatory bowel disease (IBD), and is increasingly linked to the gastrointestinal pathologies comorbid with neurodevelopmental disorders such as autism spectrum disorder (ASD) (Mitchell and Aydemir, 2025; Bjørklund, 2026). However, its role in AAC remains incompletely understood. To investigate the effects of ZnG on AAC, we established a mouse model of intestinal injury induced by antibiotics in combination with PGN, mimicking the post dysbiosis state with secondary G^+^ bacteria associated challenge, and evaluated the therapeutic potential of ZnG in promoting intestinal barrier repair.

Our findings showed that mice in the ABX+PGN group developed significant body weight loss accompanied by pronounced intestinal injury, as evidenced by worsened histopathology and clear ultrastructural abnormalities on TEM. Consistent with previous studies demonstrating that Zinc supplementation has been shown to promotes the recovery of body weight and intestinal tissue repair in various enteritis models (Hassanein et al., 2021; Zhang et al., 2024b), our study also observed that ZnG treatment facilitated body weight regain and significantly improved colonic architectural integrity in mice.

Intestinal epithelial barrier dysfunction is a central pathogenic mechanism in colitis. The goblet cell derived MUC2 is the major structural component of the colonic mucus layer and constitutes the first line of epithelial defense (Song et al., 2023), physically separating luminal contents from intestinal epithelial cells (IECs) and thereby limiting direct stimulation by pathogens and their metabolites (Okumura and Takeda, 2024). In the present study, colonic MUC2 expression was markedly reduced in the ABX+PGN group, indicating disruption of the mucus barrier, consistent with reports from multiple experimental models of ulcerative colitis (Yao et al., 2021; Zhang et al., 2024a). Previous work has shown that zinc supplementation can enhance MUC2 expression and strengthen barrier function (Liu et al., 2014). In line with these findings, we observed that ZnG significantly attenuated the ABX+PGN induced down regulation of MUC2, suggesting that its protective effect may, at least in part, be mediated by preservation of mucus barrier integrity.

Beyond the mucus layer, TJs are a key structural determinant of intestinal epithelial barrier integrity. By forming an apical junctional complex that creates a sealing effect, TJs restrict paracellular infiltration of luminal noxious substances (Macura et al., 2024). TJs are primarily composed of transmembrane proteins, including Occludin and claudin, together with scaffold proteins (ZO-1) (Furuse, 2010). TJ disruption is well-recognized feature in several intestinal disorders, particularly in IBD. Although the underlying mechanisms remain incompletely understood, TJ dysfunction is considered a central component of disease progression (Horowitz et al., 2023). Zinc deficiency has been frequently reported in patients with IBD and infectious diarrhea, suggesting a potential association between zinc imbalance and impaired epithelial barrier homeostasis (Mitchell and Aydemir, 2025). Previous studies have reported that a high Zinc diet increases Occludin and ZO-1 expression and reduces intestinal permeability in weaned piglets (Zhang and Guo, 2009). In the present study, we further demonstrated that ZnG supplementation markedly restored the ABX and PGN induced downregulation of ZO-1 and Occludin in the colonic tissue of mice, supporting a protective effect of ZnG on the mechanical barrier in AAC.

In addition, DAO is widely used as a sensitive biochemical marker of intestinal mucosal injury and alterations in barrier integrity, and is therefore applied as an indirect indicator of changes in intestinal permeability (Lyu et al., 2025). Our results showed that circulating DAO levels were significantly increased in mice following PGN stimulation, whereas ZnG intervention effectively attenuated this elevation. This finding is consistent with our previous clinical observations: in a study of ZnG combined with probiotics for pneumonia associated AAD, plasma DAO levels decreased significantly after ZnG treatment in pediatric patients (Xiang et al., 2019). Taken together, the animal and clinical data suggest that ZnG may confer protection against intestinal barrier injury, potentially by reducing intestinal permeability.

Previous studies have shown that administration of PGN derived from *S. aureu* can trigger intestinal inflammation and injury in mice and rats via activation of the NF-κB pathway (Wang et al., 2004; Fokam et al., 2020). In the present study, PGN treatment upregulated the expression of its receptor TLR2 and increased p65 expression in colonic tissues, indicating activation of the TLR2/NF-κB signaling pathway. Accumulating evidence suggests that zinc is not merely an essential micronutrient but also functions as an intracellular signaling molecule involved in immune regulation and inflammatory control. Zinc has been shown to suppress canonical NF-κB activation through multiple pathways, including induction of the zinc finger protein A20 (TNFAIP3), a key endogenous inhibitor that terminates NF-κB activation by restricting upstream signaling cascades (Jarosz et al., 2017). In addition, zinc has been reported to reduce oxidative stress, and suppress the production of pro-inflammatory cytokines, thereby contributing to the maintenance of intestinal immune homeostasis (Mitchell and Aydemir, 2025). Experimental studies further support the role of zinc in modulating NF-κB mediated inflammatory responses. For example, dietary supplementation with zinc oxide (ZnO) downregulated the expression of TLR2 and several cytokines, including IL-8, IL-6, IL-22, and TGFβ1, in the intestinal mucosa of weaned piglets (Ng'ang'a et al., 2025). Similarly, zinc intervention has been reported to suppress NF-κB associated inflammatory signaling and ameliorate IBD models (Wen et al., 2024). Consistent with these findings, we observed that ZnG significantly attenuated the ABX and PGN induced upregulation of TLR2 and NF-κB. Collectively, these data suggest that ZnG may alleviate AAC by inhibiting the PGN/TLR2/NF-κB signaling axis. Nevertheless, the precise molecular mechanisms underlying ZnG mediated regulation of NF-κB signaling, including the potential involvement of A20 and other zinc responsive regulators, warrant further investigation.

An increasing body of evidence highlights macrophage polarization as a tractable target for alleviating intestinal inflammatory disorders. For instance, luteolin has been reported to ameliorate DSS induced colitis by rebalancing M1/M2 macrophage polarization (Yang et al., 2025); in addition, gene delivery approaches that reduce intestinal M1 macrophages while increasing M2 macrophages have also been shown to alleviate UC in mice (Liu et al., 2022). Meanwhile, zinc deficiency was demonstrated to increase the proportion of pro-inflammatory (M1 like) macrophages within colonic lamina propria mononuclear cells in inflamed colon (Higashimura et al., 2020), and ZnO containing nanomaterials have also been associated with a shift toward an M2 like phenotype (Meng et al., 2026). Consistent with these observations, we found that ABX and PGN challenge upregulated the M1 associated marker iNOS in colonic tissue, whereas ZnG intervention reversed this increase and further enhanced the M2 associated marker ARG-1. Together, these findings suggest that ZnG may modulate the expression of macrophage polarization associated markers, which may contribute to the alleviation of ABX and PGN induced immune imbalance.

Beyond ameliorating intestinal barrier function, we further assessed the comprehensive protective role of ZnG by evaluating its capacity to restore the gut microbial ecosystem. At the phylum level, Bacteroidetes and Firmicutes are highly abundant in the Intestinal (Schultz et al., 2017). Increased abundance of Proteobacteria has been associated with reduced production of short chain fatty acids (SCFAs) (Meng et al., 2020), leading to intestinal infections and damage (Shao et al., 2020). Consistent with this, our results showed that ABX and PGN treatment induced overgrowth of Proteobacteria while suppressing Bacteroidetes, and these changes were reversed by ZnG supplementation. One study demonstrated that ZnO nanoparticles restored intestinal barrier integrity and gut microbiota diversity, alleviating TNBS induced colitis (Yu et al., 2025). Similarly, our diversity analysis revealed declines in both α diversity and β diversity following ABX and PGN exposure, which were partially counteracted by zinc supplementation. These results indicate that ABX and PGN induced colitis disrupts protective microbial communities, whereas ZnG supplementation helps partially restore the microbial balance, thereby improving intestinal barrier function and playing a crucial role in mitigating gut inflammation.

## 5 Conclusions

This study investigated the therapeutic potential of ZnG against ABX and PGN induced intestinal injury in mice through a multidimensional mechanism involving the gut barrier, macrophage immunity, and gut microbiota regulation. The results demonstrated that ZnG significantly alleviated ABX and PGN induced adverse manifestations, including weight loss, colonic structural damage, mucus barrier disruption, and increased intestinal permeability. In addition, ZnG may modulated the expression of markers associated with macrophage polarization, thereby contributing to the regulation of mucosal immune homeostasis. Furthermore, gut microbiota analysis revealed that ZnG partially mitigated microbial dysbiosis, partially modulated microbial diversity, increased the abundance of certain beneficial phyla while inhibited the proliferation of pathogenic bacteria. Collectively, these findings suggest that ZnG exerts multilayered protective effects by repairing the physical barrier integrity, regulating immune homeostasis, and partially restoring microbial ecological balance.

## Data Availability

The 16S rRNA gene sequencing data presented in the study are deposited in the NCBI SRA repository, with the accession number PRJNA1510176.
